# A semi‐supervised domain adaptation method with scale‐aware and global‐local fusion for abdominal multi‐organ segmentation

**DOI:** 10.1002/acm2.70008

**Published:** 2025-02-09

**Authors:** Kexin Han, Qiong Lou, Fang Lu

**Affiliations:** ^1^ School of Science Zhejiang University of Science and Technology Hangzhou China

**Keywords:** abdominal multi‐organ segmentation, scale‐aware, semi‐supervised domain adaptation, transformer

## Abstract

**Background:**

Abdominal multi‐organ segmentation remains a challenging task. Semi‐supervised domain adaptation (SSDA) has emerged as an innovative solution. However, SSDA frameworks based on UNet struggle to capture multi‐scale and global information.

**Purpose:**

Our work aimed to propose a novel SSDA method to achieve more accurate abdominal multi‐organ segmentation with limited labeled target domain data, which has a superior ability to capture the multi‐scale features and integrate local and global information effectively.

**Methods:**

The proposed network is based on UNet. In the encoder part, a scale‐aware with domain‐specific batch normalization (SAD) module is integrated to adaptively extract multi‐scale features and to get better generalization across source and target domains. In the bottleneck part, a global‐local fusion (GLF) module is utilized for capturing and integrating both local and global information. They are integrated into the framework of self‐ensembling mean‐teacher (SE‐MT) to enhance the model's capability to learn common features across source and target domains.

**Results:**

To validate the performance of the proposed model, we evaluated it on the public CHAOS and BTCV datasets. For CHAOS, the proposed method obtains an average DSC of 88.97% and ASD of 1.12 mm with only 20% labeled target data. For BTCV, it achieves an average DSC of 88.95% and ASD of 1.13 mm with 20% labeled target data. Compared with the state‐of‐the‐art methods, DSC and ASD increased by at least 0.72% and 0.33 mm on CHAOS, 1.29% and 0.06 mm on BTCV, respectively. Ablation studies were also conducted to verify the contribution of each component of the model. The proposed method achieves a DSC improvement of 3.17% over the baseline with 20% labeled target data.

**Conclusion:**

The proposed SSDA method for abdominal multi‐organ segmentation has a powerful ability to extract multi‐scale and more global features, significantly improving segmentation accuracy and robustness.

## 1 INTRODUCTION

Abdominal multi‐organ segmentation is a key step in various clinical applications. However, due to the complexity nature of the anatomy, manual delineation of multi‐organ from computed tomography (CT) and magnetic resonance imaging (MRI) is both tedious and time‐consuming.^1^ Recently, deep learning techniques have brought new breakthroughs to medical image segmentation. Typically, fully convolutional networks, such as UNet,^2^ have achieved remarkable results in medical image segmentation through end‐to‐end learning.

**FIGURE 1:**
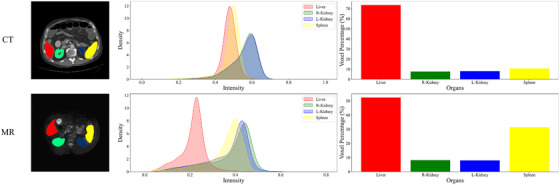
Visual and statistical comparison of unpaired multi‐modal abdominal multi‐organ.

However, training deep learning models usually requires a large amount of annotated data, while the annotation of medical image data is usually a time‐consuming and labor‐intensive task. Furthermore, a lot of easily accessible unlabeled medical image data are not fully used. Recently, semi‐supervised learning and some domain adaptation methods leverage a large amount of unlabeled data to improve learning when the labeled data are limited. Domain adaptation methods transfer the knowledge learned from one domain(source domain) to another related domain(target domain).^3^ This is vital for applications with limited annotated data in the target domain, especially for medical images. Specifically, medical images usually come from different scanners, protocols, locations, and modalities, which can lead to different data distributions. As shown in Figure 1, the first column displays spatial misalignment and scale variation issues between different modalities. The second column illustrates the intensity distribution within the segmented regions across the modalities. The third column shows the voxel percentage within each segmented region, further emphasizing the scale variation challenges between different modalities and among patients. This leads to a mismatch in data distribution, which can seriously degrade the performance of the model pre‐trained well in the target domain. Domain adaptation is essential for addressing these issues.

Semi‐supervised learning (SSL) is another promising approach to address the challenge of leveraging limited labeled data along with a larger amount of unlabeled data.^4^ This strategy enables the extraction of meaningful features from extensive unlabeled data, thus reducing the high demand for labeled data. This is especially true in the field of medical imaging, where obtaining large quantities of labeled data can be impractical or prohibitively expensive.^5^ Therefore, it is crucial to develop SSL techniques that can effectively utilize both labeled and unlabeled data to produce reliable and precise segmentation models, even with limited resources. However, the efficacy of SSL may diminish notably in scenarios where labeled images are scarce.

Recently, an integrated approach known as semi‐supervised domain adaptation (SSDA) has emerged, combining domain adaptation and SSL. By leveraging extensive open‐source data, SSDA can make it possible to achieve satisfactory outcomes with minimal annotation on some private datasets.^6^ This integrative strategy has a great advantage in overcoming the challenges associated with domain shifts, particularly in the field of medical image segmentation.^7^, ^8^ Recently, a novel paradigm for SSDA, named CS‐CADA,^9^ was introduced by incorporating cross‐domain contrastive learning and the framework of self‐ensembling mean‐teacher^10^ (SE‐MT). It was further refined through the use of semantic and stylistic contrastive learning,^11^ aiming to capture the common features more effectively between the source and target domains. However, the existing SSDA methods usually use fixed‐size convolution kernels, which limit their adaptability to various organ sizes across different modalities or within the same modality. Recently, more and more researchers have paid their attention to deep networks with multi‐scale modules.^12^ For example, the DMCNN^13^ framework integrated multi‐scale features, but it still has a challenge in preserving fine details at deeper levels due to reduced resolution. MSU‐Net^14^ employed a multi‐scale UNet architecture that ensured a balance between geometric fidelity and semantic richness, effectively capturing the intricate details necessary for complex image segmentation. Moreover, while convolution operations are effective in capturing local relationships, they may fail to capture global relationships, which may lead to the model's inability to identify different organs. As a result, traditional Convolutional Neural Networks (CNN) segmentation networks may lack comprehensive contextual information. To address this limitation, the vision transformer (ViT)^15^ is currently being used to extract extensive global contextual features in medical image segmentation tasks. Nonetheless, the original ViT module lacks spatial inductive biases,^16^ and needs a large number of parameters to effectively learn global representations. This can increase the risk of overfitting, particularly in unpaired cross‐domain segmentation tasks with limited data. Some researchers have been proposed to integrate CNN and ViT for better extracting local and global features. A framework named CoTr^17^ combines CNNs with an efficient deformable Transformer, which focuses on key positions via deformable self‐attention to reduce complexities, thus enabling effective processing of high‐resolution, multi‐scale feature maps. CoTrFuse,^18^ a network that integrates the strengths of CNNs and Transformers through dual encoders and a fusion module, brings new benchmarks in medical image segmentation.

To tackle these challenges, we introduce an SSDA framework, aimed at achieving domain adaptation for unpaired modalities and then leveraging a little labeled target data to achieve accurate abdominal multi‐organ segmentation. The contributions in this paper can be summarized as follows:

1. The scale‐aware with domain‐specific batch normalization (SAD) module integrates domain‐specific batch normalization (DSBN)^19^ and scale‐aware convolution into the encoder of the 3D UNet, improving the model's ability to capture organs of various sizes.
2. The global‐local fusion (GLF) module incorporates the CNN and ViT into the bottleneck layer, enhancing the model's ability to learn and generalize by leveraging local spatial and global context information.
3. The self‐ensembling mean‐teacher (SE‐MT)^10^ training framework effectively utilizes labeled and unlabeled data across domains with different distributions, improving model robustness and adaptability.

## 2 METHODS

Let S and T respectively denote the source and target domains images, which can be either 3D CT or MRI images depending on the direction of domain adaptation being performed. The training data contains three subsets, a labeled subset from source domain $S_{L}={\left\{\left(x_{i}^{S_{L}},y_{i}^{S_{L}}\right)\right\}}_{i=1}^{n^{S_{L}}}$, a limited labeled subset from target domain $T_{L}={\left\{\left(x_{i}^{T_{L}},y_{i}^{T_{L}}\right)\right\}}_{i=1}^{n^{T_{L}}}$ and a lager unlabeled subset from target domain $T_{U}={\left\{x_{i}^{T_{U}}\right\}}_{i=1}^{N^{T_{U}}}$.

**FIGURE 2:**
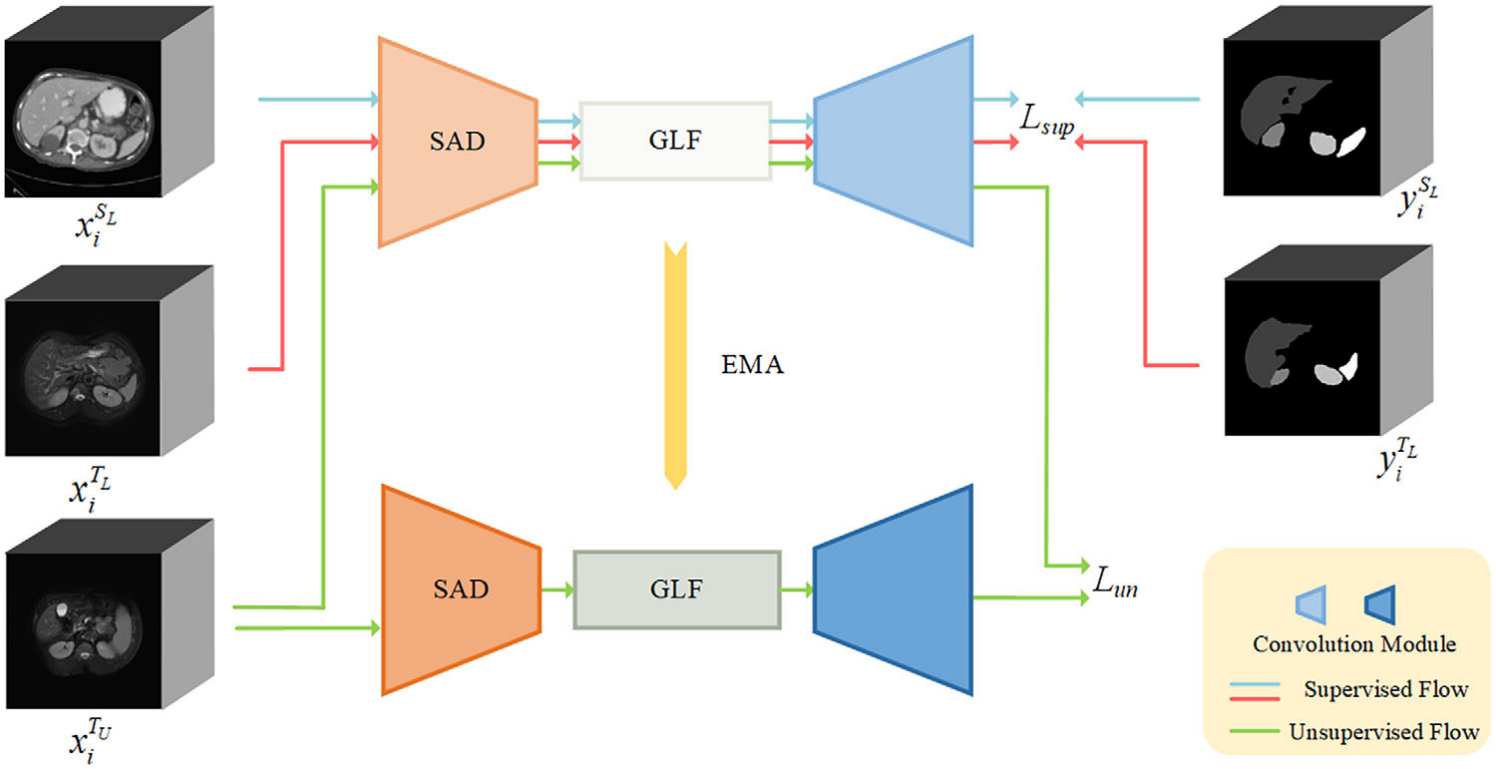
Flowchart of the proposed abdominal multi‐organ segmentation method.

The proposed method consists of three main parts: (1) a SE‐MT structure enhances the consistency and reliability of the segmentation outcomes; (2) a SAD module integrates scale‐aware convolutions with DSBN in the segmentation network's encoder; and (3) a GLF module integrates CNN and ViT to extract local and global contextual information in the bottleneck. The flowchart of the proposed method is depicted in Figure 2.

### 2.1 Scale‐aware with DSBN (SAD) module

**FIGURE 3:**
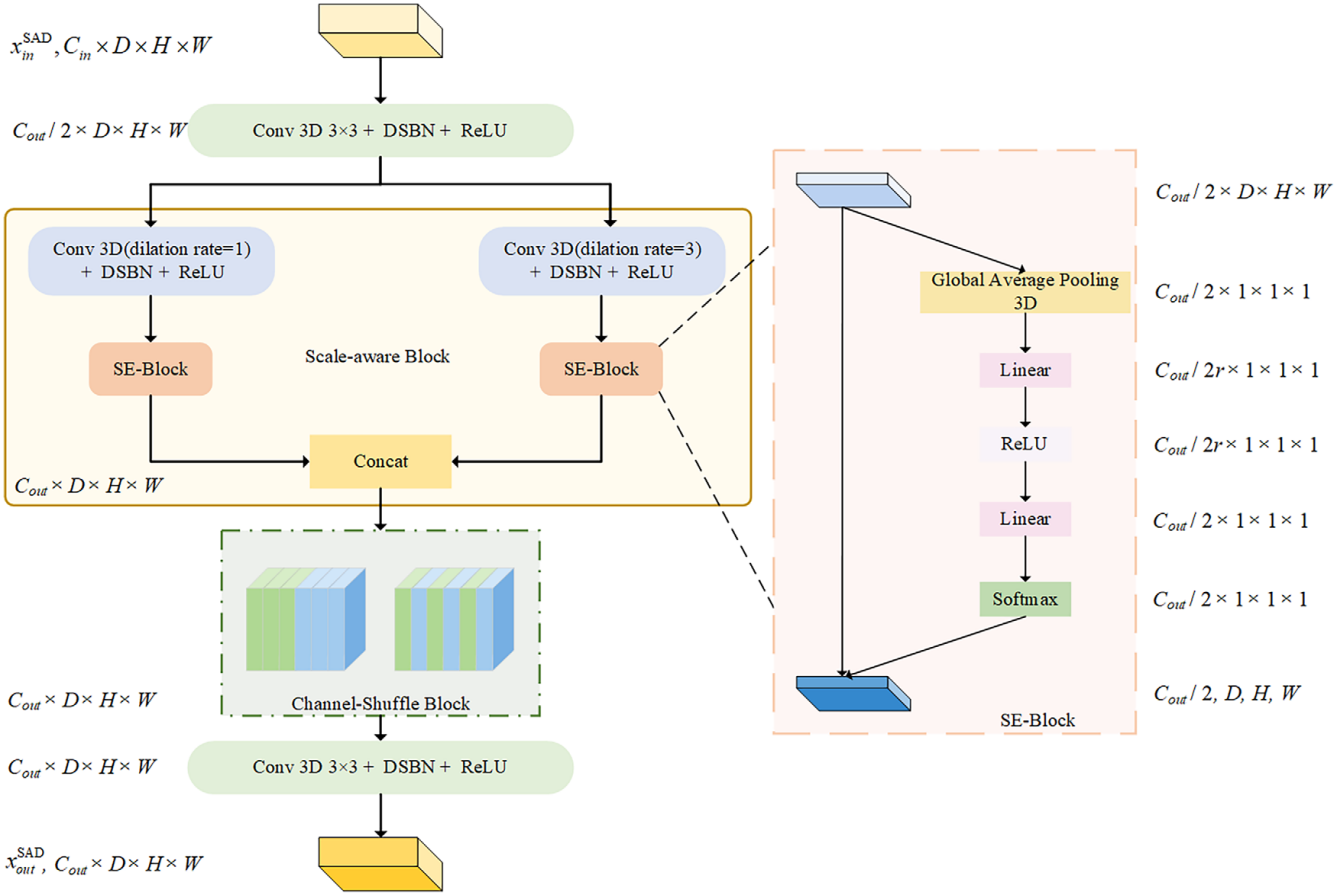
The structure of the domain‐specific batch normalization (SAD) module.

Variations in scale and discrepancies in intensity distribution across different imaging modalities make the accuracy of segmentation decline. To solve this problem, an SAD module is proposed, and Figure 3 displays its structure in details.

The SAD module is structured with two convolution blocks, each employing DSBN for normalization (Conv‐D), complemented by a scale‐aware block and a channel‐shuffle block ($f_{shuffle}$), ^20^ as detailed below:

(1) $$x_{\mathrm{out}}^{\mathrm{SAD}}=\mathrm{Conv}-D\left(f_{\mathrm{shuffle}}\left(\mathrm{SAD}\left(\mathrm{Conv}-D\left(x_{\mathrm{in}}^{\mathrm{SAD}}\right)\right)\right)\right)$$

The input tensor $x_{in}^{\mathrm{SAD}}\in R^{B\times C_{in}\times D\times H\times W}$ initially passes through a Conv‐D layer, and then through two distinct convolutional branches. They both utilize a kernel size of 3 × 3, but with different dilation rates of 1 and 3. Then, each convolutional branch is augmented with an SE‐Block^21^ to reinforce important channel features and weaken less crucial ones, optimizing performance outcomes. Moreover, we concatenate the two recalibrated multi‐scale tensors and apply a channel shuffle operation to facilitate interaction between multi‐scale features.

Traditional batch normalization techniques assume a consistent intensity distribution across different domains, which can lead to a bad performance. It cannot effectively account for the shift in intensity distributions between source and target domains.

To overcome this limitation, we adopt the DSBN approach. It ensures that features from each domain are normalized based on their own statistics.

For a given domain d, the DSBN processes the input features $x_{d}$ for each feature map (or channel) independently, employing a unique set of normalization parameters for each. This approach results in the domain‐specific normalized features ${\tilde{y}}_{d}$ for each channel as follows:

(2) $${\tilde{y}}_{d}=\frac{x_{d}-\mu_{d}}{\sqrt{\sigma_{d}+\delta }}\;$$

where, δ is a small constant added to prevent division by zero, $\mu_{d}$ and $\sigma_{d}$ are the mean and variance of the domain‐specific features within a mini‐batch.

Subsequently, a domain‐adaptive affine transformation is applied, characterized by the scale parameter $\kappa_{d}$ and the shift parameter $\vartheta_{d}$, leading to the final adjusted output $y_{d}$:

(3) $$y_{d}=\kappa_{d}\;\cdot {\tilde{y}}_{d}+\vartheta_{d}$$

By adopting DSBN, our model can handle the inherent variability between domains, achieving better generalization across different data distributions.

With wide‐scale variability and variable intensity distributions across multi‐modalities in abdominal multi‐organ images, accurate segmentation becomes more complex. The SAD Module addresses these issues by capturing and contextualizing information at multiple scales. It enhances the model's ability to accurately identify and characterize anatomical features, ensuring precise segmentation.

### 2.2 Global‐local fusion (GLF) module

**FIGURE 4:**
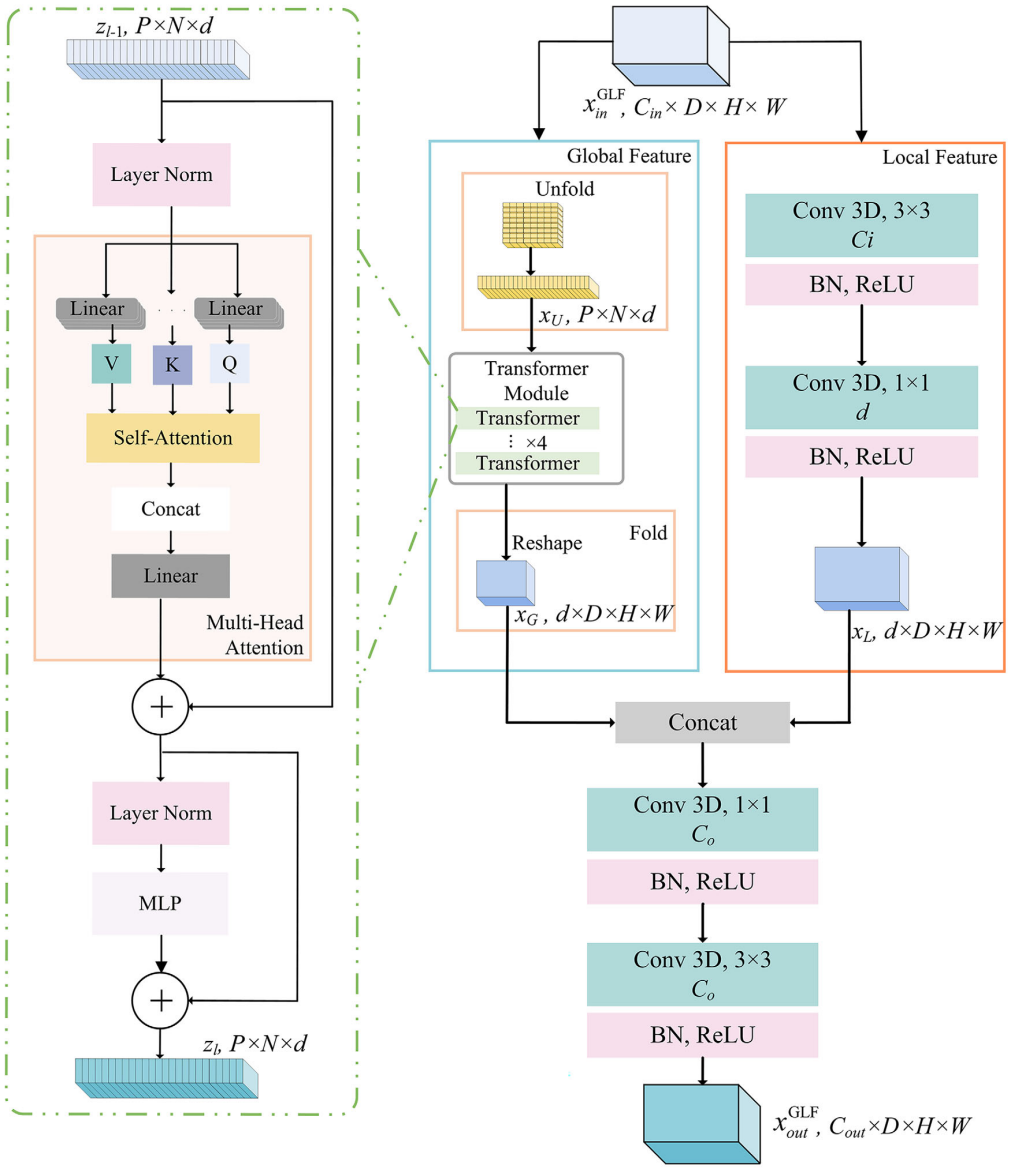
The structure of global‐local fusion (GLF) module.

The current SSDA methods usually use the same convolution kernels to extract invariant representations across different modalities. However, the convolution operations usually fail to capture the global contextual details and thus reduce the segmentation result. The proposed method aims to combine local spatial features and global contextual features to obtain more broadly applicable representations across various modalities. To achieve this, we introduce the GLF module, as is shown in Figure 4.

In the GLF module, we denoted the initial input tensor as $x_{in}^{\mathrm{GLF}}\in R^{B\times C_{in}\times D\times H\times W}$. It is processed through the local representations module and the global representations module in parallel. The local representations module employs a sequence of convolutional layers, specifically a 3 × 3 and a 1 × 1 convolutional layer. It yields a tensor $x_{L}\in R^{B\times d\times D\times H\times W}$, with $d=\frac{C_{in}}{2}$. Meanwhile, the global representations module unfolds the input tensor by convolution operations. This novel approach more effectively captures spatial induction biases compared to the linear projection of flattened patches used in traditional ViT modules. The tensor $x_{U}\in R^{B\times P\times N\times d}$ is obtained by unfolding, where $P=p_{d}\;\times p_{w}\times p_{h}$ denotes the patch size and $N=\frac{D\times H\times W}{P}$ denotes the total number of patches, with all dimensions $p_{d}$, $p_{w}$, and $p_{h}$ set to 2 for optimized GPU memory use. The tensors from both pathways are then concatenated to form a comprehensive representation that incorporates both detailed local features and a broader global context. For global context learning, a lightweight transformer module without positional embeddings processes the tensor $x_{U}$. The output tensor of the global representations module, $x_{G}\in R^{B\times d\times D\times H\times W}$, can be described as follows:

(4) $$x_{G}=f_{\mathrm{Fold}}\;\left(f_{\mathrm{Trans}}\left(x_{U}\right)\right)$$

where $f_{Fold}$ is the tensor reshaping fold operation, and $f_{Trans}$ denotes the transformer module encompassing four transformer layers. The output $z_{l}\in R^{B\times P\times N\times d}$ of each transformer layer (l, where 0<l≤L) results from the multi‐head attention (MHA) and multi‐layer perceptron (MLP) networks, processing the preceding layer's output $z_{l-1}\in R^{B\times P\times N\times d}$, as formulated in:

(5) $$z_{l}^{\prime }=\mathrm{MHA}\left(\mathrm{LN}\left(z_{l-1}\right)\right)+z_{l-1}$$

(6) $$z_{l}=\mathrm{MLP}\left(\mathrm{LN}\left(z_{l}^{\prime }\right)\right)+z_{l}^{\prime }$$

Here, $z\in R^{B\times P\times N\times d}$ denotes the input tensor, $d_{head}=8$ the dimension per SA head, and $Q_{i}$, $K_{i}$, $V_{i}$ are derived from z through linear transformations, with $Q_{i}$, $K_{i}$, $V_{i}\in R^{B\times P\times N\times d_{head}}$. Thus, the MHA module is expressed as:

(7) $$SA_{i}\;\left(z\right)=\mathrm{softmax}\left(\frac{Q_{i}\cdot {\left(K_{i}\right)}^{⊤}}{\sqrt{d_{\mathrm{head}}}}\right)\cdot V_{i}$$

Here, denotes the input tensor, $d_{head}=8$ the dimension per SA head, and $Q_{i}$, $K_{i}$, $V_{i}$ are derived from z through linear transformations, with. Thus, the MHA module is expressed as:

(8) $$\mathrm{MHA}\;\left(z\right)=\left[SA_{1}\left(z\right);\cdots ;SA_{n_{\mathrm{head}}}\left(z\right)\right]\;W_{\mathrm{linear}}$$

where the concatenation operation is denoted by [⋯], and $W_{linear}\in R^{B\times (n_{head}\cdot d_{head})\times d}$ symbolizes the linear transformation layer's trainable weights. Finally, shared convolutional layers are utilized to transition $x_{G}\in R^{B\times d\times D\times H\times W}$ into the output tensor $x_{out}^{\mathrm{GLF}}\in R^{B\times C_{out}\times D\times H\times W}$, which showcases enhanced modality‐invariant representations with global contexts in comparison to outputs derived from other methods’ shared convolutional bottleneck layers.

Given pairs of images and annotations $\left(x_{i}^{S_{L}},y_{i}^{S_{L}}\right)\in S_{L}$ from the source domain S and $\left(x_{i}^{T_{L}},y_{i}^{T_{L}}\right)\in T_{L}$ from the target domain T, we formulate a loss function to simultaneously refine these parameter sets:

(9) $$L_{\sup}=\sum_{i=1}^{n^{S_{L}}}L_{\mathrm{seg}}\;\left(p_{i}^{S_{L}},y_{i}^{S_{L}}\right)+\sum_{i\;=1}^{n^{T_{L}}}L_{\mathrm{seg}}\left(p_{i}^{T_{L}},y_{i}^{T_{L}}\right)$$

where the predicted outputs for source and target domain images, $p_{i}^{S_{L}}=\psi \left(x_{i}^{S_{L}};\Theta^{S}\right)$ and $p_{i}^{T_{L}}=\psi \left(x_{i}^{T_{L}};\Theta^{T}\right)$, are obtained through the models parameterized by $\Theta^{S}$ and $\Theta^{T}$, respectively. Here, $L_{sup}$ represents a composite segmentation loss, incorporating both the cross‐entropy and dice loss components, aimed at enhancing segmentation performance across domains.

### 2.3 Self‐ensembling mean teacher (SE‐MT) framework

To effectively mitigate data distribution discrepancies across different modalities, we implement the SE‐MT strategy.^10^ It has significant advantages in scenarios where the target domain has a limited amount of labeled data and a substantial amount of unlabeled data.

The SE‐MT framework is based on the Mean Teacher^22^ architecture, which comprises a student model and a teacher model. The student model is trained directly with gradient descent, and the teacher model is an exponential moving average (EMA) of the student model's parameters.

(10) $$\Theta_{k}^{T^{\prime }}={\eta \Theta }_{k-1}^{T^{\prime }}+\left(1-\eta \right)\Theta_{k}^{T}$$

where η denotes the decay coefficient for the EMA, constrained to the interval [0, 1]. The parameters $\Theta_{k}^{T}$ represent the current parameters of the student model. EMA is designed to stabilize the learning process by incorporating the historical knowledge accumulated by the teacher model, which in turn provides more accurate guidance for the student model, especially when handling unlabeled data.

The consistency loss $L_{un}$ is a critical component, ensuring that the student and teacher models produce similar outputs for the same input under different noise Δ and $\Delta^{\prime }$. It is defined as:

(11) $$L_{\mathrm{un}}=\sum_{i=1}^{n^{T_{U}}}L_{\mathrm{mse}}\;\left(\psi \left(x_{i}^{T_{U}};\Theta^{T},\Delta \right),\psi \left(x_{i}^{T_{U}};{\left(\Theta^{T}\right)}^{\prime },\Delta^{\prime }\right)\right)$$

Here, $\psi (x_{i}^{T_{U}};\Theta^{T},\Delta )$ and 
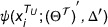
 denote the predictive functions of the student and teacher models, respectively. $x_{i}^{T_{U}}$ represents an unlabeled sample from the target domain, and $\Theta^{T}$ and 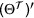 are the parameters of the student and teacher models, respectively. The mean squared error loss $L_{mse}$ is employed to compute the distance between the predictions of the student and teacher models.

In summary, by combining the supervised loss $L_{sup}$ as defined in Equation (9) with the unsupervised consistency loss $L_{un}$ detailed in Equation (11), the overall training loss for the proposed framework is as follows:

(12) $$L_{\mathrm{total}}=L_{\sup}+\lambda \cdot L_{\mathrm{un}}$$

Here, λ balances the contributions of supervised and unsupervised losses during the optimization process. The supervised component of the loss is constituted by the dice loss and the cross‐entropy loss. These are modulated by the parameters $\lambda_{dice}$ and $\lambda_{ce}$, respectively, allowing for precise adjustment to the training process. Upon the completion of the training phase, the system procures the segmentation of abdominal multi‐organ by executing a forward pass through the student network, which is parameterized by $\Theta^{T}$.

### 2.4 Experimental setup

#### 2.4.1 Datasets and evaluation metrics

In our study, we utilize two public datasets for 3D abdominal multi‐organ abdominal segmentation: 30 CT volumes from the BTCV dataset^23^ and 20 T2‐SPIR MRI volumes from the ISBI 2019 CHAOS Challenge.^24^ To ensure a fair comparison with existing unsupervised domain adaptation (UDA) approaches,^25^ we also use the same publicly available datasets. We focus on the liver, right kidney, left kidney, and spleen, which are consistently annotated across both datasets. The slice thickness ranges between 7.0 and 8.0 mm in the CHAOS dataset and 2.5 and 5.0 mm in the BTCV dataset. For the original abdominal dataset, MRI volumes only include the abdomen area, whereas CT volumes include the region from the neck to the knee. In the preprocessing phase, we first crop the CT and MRI scans, aiming to ensure that both CT and MRI scans cover only the structures or organs targeted for segmentation. Then, we resample all volumes to a uniform dimension of 256 × 256 × 32. Next, we limit the pixel values of the CT images to the range of [−350, 350]. Finally, we normalize the voxel values of both CT and MRI images to a range of [0,1], ensuring uniform input for our segmentation models.

For model training and evaluation, 80% of the scans from both datasets are allocated for training, resulting in 24 CT scans and 16 MRI scans designated for the training set. The remaining 20% are reserved for testing, comprising six CT scans and four MRI scans. To enhance the precision of our models and mitigate overfitting, we apply some online data augmentation strategies by MONAI^26^ on the training datasets, such as rotation, affine and contrast adjustment. Specifically, for each training iteration, we dynamically generate new samples through transformations including random rotations within a range of −30° to +30°, random affine transformations within shearing from −0.1 to +0.1 (in radians), and contrast adjustments with a random gamma value between 1.5 and 2. These strategies help diversify the input data and effectively alleviate the limitations associated with a relatively small dataset.

In order to evaluate the segmentation performance of the model, common evaluation metrics such as the dice similarity coefficient (DSC) and the average symmetric surface distance (ASD) are employed. Model performance is superior when the DSC is higher, and the ASD values are lower.

#### 2.4.2 Implementation details

Our network is implemented by using the PyTorch framework and MONAI for data enhancement. All experimental work is carried out on a workstation running Ubuntu 20.04, equipped with two 24G NVIDIA GeForce RTX 3090 GPUs and an Intel Xeon Gold 6226 CPU at 2.90 GHz. The batch size is set to eight, comprising four images from $S_{L}$, two from $T_{L}$, and two from $T_{U}$. Furthermore, segmentation networks are trained iteratively over 200 epochs using AdamW optimizers, starting with an initial learning rate of 0.0009 and a weight decay rate of 0.00005. The learning rate is adjusted to decay exponentially at a rate of 0.95 every 250 iterations. In our mean‐teacher backbone, we adopted the UNet^2^ architecture for segmentation tasks and enhanced its encoder and bottleneck sections with the SAD module and GLF module, respectively. The EMA decay rate is determined through empirical testing to be 0.99.^27^ To dynamically adjust the hyperparameter η, a time‐dependent Gaussian warming‐up function is employed, represented by the equation $\eta \;\left(k\right)=0.1e^{-5{\left(1-\frac{k}{k_{max}}\right)}^{2}}$. In this equation, k denotes the current iteration of training, and $k_{max}$ signifies the final iteration.

## 3 RESULTS AND DISCUSSION

### 3.1 Comparison with other state‐of‐the‐art methods

To illustrate the efficiency of our method in addressing abdominal multi‐organ domain adaptation, we conduct a comparative analysis against a baseline model that utilizes only $T_{L}$. The baseline of our method consists of a modified UNet, augmented with SAD module and GLF module in the encoder and bottleneck sections, respectively, referred to as Supervised only. We conduct a comparative analysis using several state‐of‐the‐art techniques across four distinct categories: (1) UDA methods that utilize labeled source data, $S_{L}$, and unlabeled target data, $T_{U}$. We examined two UDA techniques, including CycleGAN^28^ and CyCADA^29^; (2) Strategies for supervised domain adaptation (SDA), which typically utilize both labeled source domain data $S_{L}$ and labeled target domain data $T_{L}$ for training. One prominent approach is fine‐tuning,^30^ in which a model is initially pre‐trained on $S_{L}$ and subsequently fine‐tuned using $T_{L}$. Additionally, the DSBN^19^ method employs separate batch normalization parameters for each domain to joint training. (3) SSL approaches that utilize both $T_{L}$ and $T_{U}$ for training, including advanced methods such as SE‐MT^10^ and UA‐MT^31^; and 4) an SSDA technique, specifically CS‐CADA.^9^ It uses $S_{L}$, $T_{L}$, and $T_{U}$. To our best knowledge, there is no other comparable 3D‐based UDA methods that were developed for abdominal multi‐organ datasets. We modified the 2D model in its open‐source code to a 3D model for CS‐CADA's experiments and used the 3D model as the backbone in other methods’ experiments (besides ours) for fairness.

**FIGURE 5:**
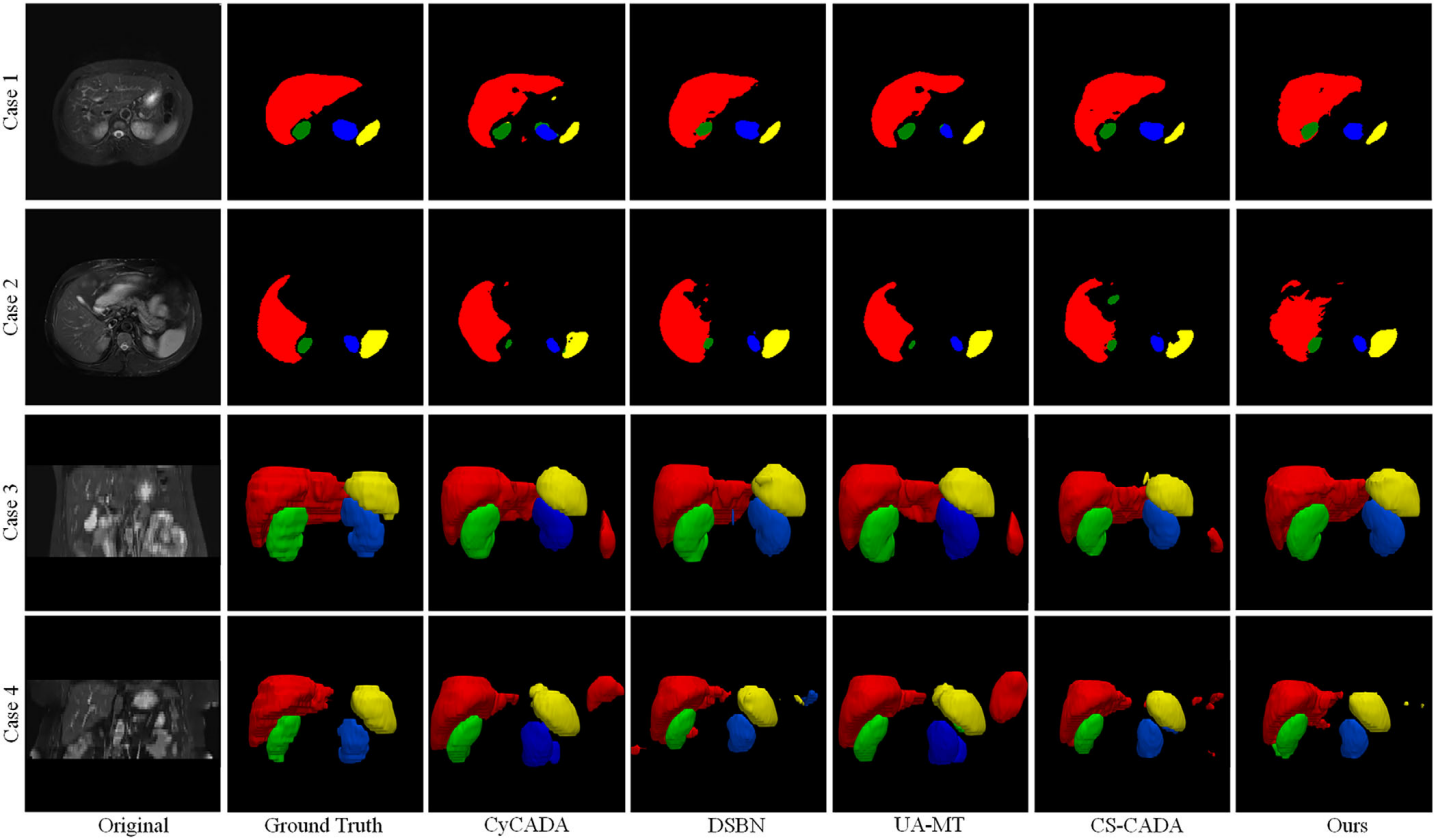
Comparative visualization of magnetic resonance imaging (MRI) segmentation in domain adaptation from computed tomography (CT) to MRI. The liver, the right kidney, the left kidney, and the spleen correspond to red, green, blue, and yellow, respectively.

**FIGURE 6:**
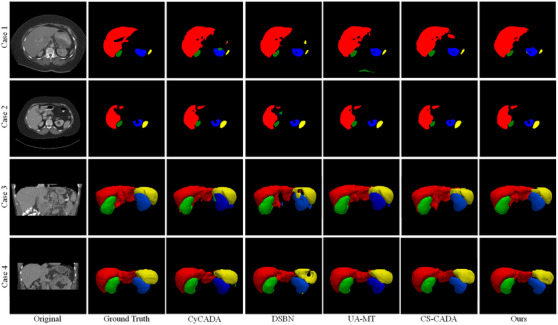
Comparative visualization of computed tomography (CT) segmentation in domain adaptation from magnetic resonance imaging (MRI) to CT. The liver, the right kidney, the left kidney, and the spleen correspond to red, green, blue, and yellow, respectively.

Tables 1 and 2 present the results of our experiments in domain adaptation from CT to MRI images and from MRI to CT images, respectively. For SDA, SSL, SSDA, and the proposed methods, we use only 20% annotated target images. We display the 2D and 3D predicted segmentation results of the proposed method and several other methods, as demonstrated in Figure 5 for CT to MRI and Figure 6 for MRI to CT. The results from the Supervised approach, which only uses labeled target domain images $T_{L}$, yielding average DSC of 75.80% and 70.87%, average ASD of 2.32 and 2.07 mm for MRI and CT, respectively. It reveals the constraints of relying on a minimal amount of labeled target data for achieving precise outcomes. UDA strategies demonstrated superior performance over Supervised‐only, and the effectiveness of CyCADA^29^ is slightly better than CycleGAN.^28^ We present the predicted segmentation results of CyCADA in Figures 5 and 6. As shown in Figure 5, the MRI segmentation results of the left kidney exhibit notable errors in Cases 1 and 4. As a result, the average DSC for the left kidney is only 78.12%, as reported in Table 1. The CT segmentation results have the same situation in Cases 1 and 3 as shown in Figure 6, with an average left kidney DSC of 80.27% and 1.33 mm for ASD in Table 2. Among the SDA techniques, DSBN^19^ obtains the best DSC and ASD score. However, the DSBN method, as illustrated for Cases 2 and 4 in Figure 5 and Cases 3 and 4 in Figure 6, also obtains a poor segmentation of the spleen. As a result, the average DSC for the spleen is only 82.63%, 79.94%, and ASD score reaches 3.58 mm, and 2.97 mm in Tables 1 and 2, respectively. However, Fine‐tuning^30^ method falls short when compared to both UDA and Supervised‐only, indicating that a small portion of labeled target data is inadequate for effectively updating the parameters of a network pre‐trained on the source domain in traditional fine‐tuning strategies. The SSL methods surpassed UDA and fine‐tuning approaches for MRI and CT datasets. However, almost all SSL methods fell short of DSBN's performance, with the notable exception of UA‐MT, which outperformed DSBN in the domain adaptation from MRI to CT with a DSC of 87.66%. The UA‐MT method, as seen in Figure 5 for MRI Cases 3 and 4, is particularly hindered by noisy backgrounds, leading to over‐segmentation. This adversely affects the MRI segmentation performance, with a total average DSC of 84.08% and an ASD of 4.10 mm. Rather than choosing between using only unlabeled target domain data or abundant labeled source domain data, the SSDA approach employs both. Within the context of CT‐MRI and MRI‐CT domain adaptation, CS‐CADA achieved an average DSC of 88.25% and 86.60%, and an average ASD of 2.42 and 1.19 mm, respectively. Like UA‐MT, CS‐CADA is also confused of complex backgrounds, and it doesn't perform well enough in MRI Cases 2, 3, 4, and CT Case 1. The proposed method achieves the best average scores across four organs both in MRI and CT segmentation. On the CHAOS dataset, the DSC score is 88.97%, and the ASD is 1.12 mm. On the BTCV dataset, the DSC score is 88.95%, and the ASD is 1.13 mm. Our method shows fewer incorrect segmentation results than others, and the results of the proposed method closely align with the ground truth. It is noteworthy that while our method's ASD for the liver remains a challenge, it achieves the second‐best result of 1.32 mm among all evaluated methods. Overall, the average DSC and ASD across the four organs of our method outperform other methods. Statistical analyses in this study were conducted using two‐sided paired *t*‐tests on the average results across four organs. It is acknowledged that individual organ‐level performance may vary. All subsequent statistical comparisons adhere to this. In Tables 1 and 2, the proposed method achieves significant improvements over other methods, with *p*‐values being at least ≤ 0.1.

**TABLE 1:** Comparison with the state‐of‐the‐art methods in domain adaptation from CT to MRI.

DSC (%)							
		Liver	R. kidney	L. kidney	Spleen	Avg.	*p*‐values
Supervised only		70.28 ± 5.21	79.73 ± 3.65	79.59 ± 4.16	73.61 ± 3.44	75.80 ± 4.73	7.00E‐03
UDA	CycleGAN^28^	88.78 ± 3.05	87.31 ± 4.08	76.75 ± 5.34	79.43 ± 8.36	83.07 ± 4.55	4.60E‐02
	CyCADA^29^	88.67 ± 3.71	89.34 ± 1.57	78.12 ± 3.69	80.17 ± 7.25	84.08 ± 3.04	1.50E‐02
SDA	Fine‐tuning^30^	60.94 ± 18.83	79.76 ± 9.71	71.58 ± 10.26	72.70 ± 13.10	71.25 ± 5.63	1.00E‐03
	DSBN^19^	**90.28 ± 3.35**	**90.68 ± 1.24**	87.79 ± 3.15	82.63 ± 7.83	87.85 ± 3.81	5.50E‐02
SSL	SE‐MT^10^	83.52 ± 4.19	87.63 ± 1.92	85.04 ± 1.52	84.19 ± 4.85	85.09 ± 1.88	3.00E‐03
	UA‐MT^31^	83.17 ± 2.68	87.34 ± 3.01	85.18 ± 3.37	80.64 ± 8.97	84.08 ± 2.86	2.10E‐02
SSDA	CS‐CADA^9^	86.87 ± 2.54	88.36 ± 1.28	89.31 ± 4.43	88.45 ± 3.17	88.25 ± 1.51	2.90E‐02
Ours		86.09 ± 3.66	88.73 ± 3.74	**90.88 ± 1.42**	**90.16 ± 2.18**	**88.97 ± 1.33**	**–**

ASD (mm)							
		Liver	R. kidney	L. kidney	Spleen	Avg.	*p*‐values
Supervised only		2.09 ± 1.83	1.63 ± 1.28	2.52 ± 1.56	3.03 ± 2.73	2.32 ± 0.58	4.90E‐02
UDA	CycleGAN^28^	2.01 ± 1.76	3.23 ± 2.96	1.92 ± 1.58	2.55 ± 1.76	2.43 ± 1.09	9.00E‐03
	CyCADA^29^	1.46 ± 1.10	1.67 ± 1.33	1.27 ± 0.53	1.61 ± 0.84	1.45 ± 0.13	7.60E‐02
SDA	Fine‐tuning^30^	3.16 ± 2.87	3.72 ± 3.23	4.14 ± 3.56	1.98 ± 1.73	3.25 ± 1.50	2.10E‐02
	DSBN^19^	**1.04 ± 0.21**	**1.01 ± 0.63**	1.23 ± 1.07	3.58 ± 3.04	1.72 ± 0.79	9.40E‐02
SSL	SE‐MT^10^	5.50 ± 3.60	8.08 ± 5.64	2.89 ± 1.87	3.89 ± 3.06	5.09 ± 1.40	6.00E‐03
	UA‐MT^31^	5.75 ± 4.56	3.63 ± 3.69	1.74 ± 1.19	5.29 ± 5.11	4.10 ± 1.65	1.80E‐02
SSDA	CS‐CADA^9^	4.61 ± 4.07	2.64 ± 2.47	1.64 ± 1.39	**0.77 ± 0.18**	2.42 ± 0.74	1.10E‐02
Ours		1.32 ± 0.28	1.38 ± 0.97	**0.55 ± 0.06**	1.24 ± 1.28	**1.12 ± 0.49**	**–**

*Note*: Best results are in bold and suboptimal results are in underlined.

Abbreviations: ASD, average symmetric surface distance; DSC, dice similarity coefficient; SDA, supervised domain adaptation; SSDA, semi‐supervised domain adaptation; SSL, semi‐supervised learning; UDA, unsupervised domain adaptation.

**TABLE 2:** Comparison with the state‐of‐the‐art methods in domain adaptation from MRI to CT.

DSC (%)							
		Liver	R. kidney	L. kidney	Spleen	Avg.	*p*‐values
Supervised only		82.44 ± 9.21	62.47 ± 36.19	69.31 ± 30.44	69.24 ± 33.68	70.87 ± 27.59	2.20E‐02
UDA	CycleGAN^28^	83.42 ± 11.39	79.28 ± 12.68	79.43 ± 17.90	77.32 ± 14.37	79.86 ± 6.54	5.30E‐02
	CyCADA^29^	84.53 ± 8.07	78.61 ± 9.61	80.27 ± 10.46	76.94 ± 18.86	80.09 ± 8.22	3.20E‐02
SDA	Fine‐tuning^30^	81.97 ± 13.22	42.68 ± 28.63	65.03 ± 20.56	73.15 ± 17.24	65.71 ± 22.06	2.04E‐01
	DSBN^19^	93.49 ± 2.11	84.58 ± 8.40	84.78 ± 6.34	79.94 ± 16.39	85.69 ± 4.87	2.60E‐02
SSL	SE‐MT^10^	92.16 ± 2.00	78.62 ± 11.30	84.22 ± 7.24	86.55 ± 7.50	85.39 ± 5.58	2.30E‐02
	UA‐MT^31^	93.08 ± 1.10	83.57 ± 8.34	85.17 ± 6.79	**88.82 ± 5.59**	87.66 ± 4.71	9.00E‐02
SSDA	CS‐CADA^9^	93.36 ± 1.35	**87.24 ± 3.71**	86.01 ± 7.18	79.77 ± 15.95	86.60 ± 6.23	4.80E‐02
Ours		**93.52 ± 0.77**	86.67 ± 6.48	**89.29 ± 2.51**	86.32 ± 6.97	**88.95 ± 3.17**	**–**

ASD (mm)							
		Liver	R. kidney	L. kidney	Spleen	Avg.	*p*‐values
Supervised only		1.52 ± 0.75	3.18 ± 2.90	1.83 ± 1.56	1.74 ± 0.82	2.07 ± 1.29	4.40E‐02
UDA	CycleGAN^28^	1.76 ± 1.55	**1.34 ± 0.98**	1.21 ± 0.90	1.94 ± 1.02	1.56 ± 1.33	1.80E‐02
	CyCADA^29^	2.63 ± 1.24	1.39 ± 0.83	1.33 ± 1.09	1.85 ± 0.96	1.80 ± 1.54	7.20E‐02
SDA	Fine‐tuning^30^	1.44 ± 1.32	5.01 ± 7.36	6.27 ± 5.82	3.08 ± 2.73	3.95 ± 3.74	4.90E‐02
	DSBN^19^	0.68 ± 0.19	1.34 ± 0.96	2.30 ± 1.67	2.97 ± 3.29	1.82 ± 1.09	5.30E‐02
SSL	SE‐MT^10^	1.21 ± 1.11	17.14 ± 17.37	2.60 ± 2.07	2.37 ± 2.40	5.83 ± 5.20	5.30E‐02
	UA‐MT^31^	0.89 ± 0.53	7.44 ± 10.22	2.55 ± 2.20	**0.63 ± 0.32**	2.88 ± 2.86	8.50E‐02
SSDA	CS‐CADA^9^	1.06 ± 0.51	1.56 ± 1.99	1.18 ± 0.96	0.96 ± 0.61	1.19 ± 0.91	6.00E‐03
Ours		**0.61 ± 0.14**	1.94 ± 2.36	**0.96 ± 0.68**	1.01 ± 0.61	**1.13 ± 0.89**	**–**

*Note*: Best results are in bold and suboptimal results are in underlined.

Abbreviations: ASD, average symmetric surface distance; DSC, dice similarity coefficient; SDA, supervised domain adaptation; SSDA, semi‐supervised domain adaptation; SSL, semi‐supervised learning; UDA, unsupervised domain adaptation.

### 3.2 Ablation study

#### 3.2.1 Results under different proportions of labeled target data

**FIGURE 7:**
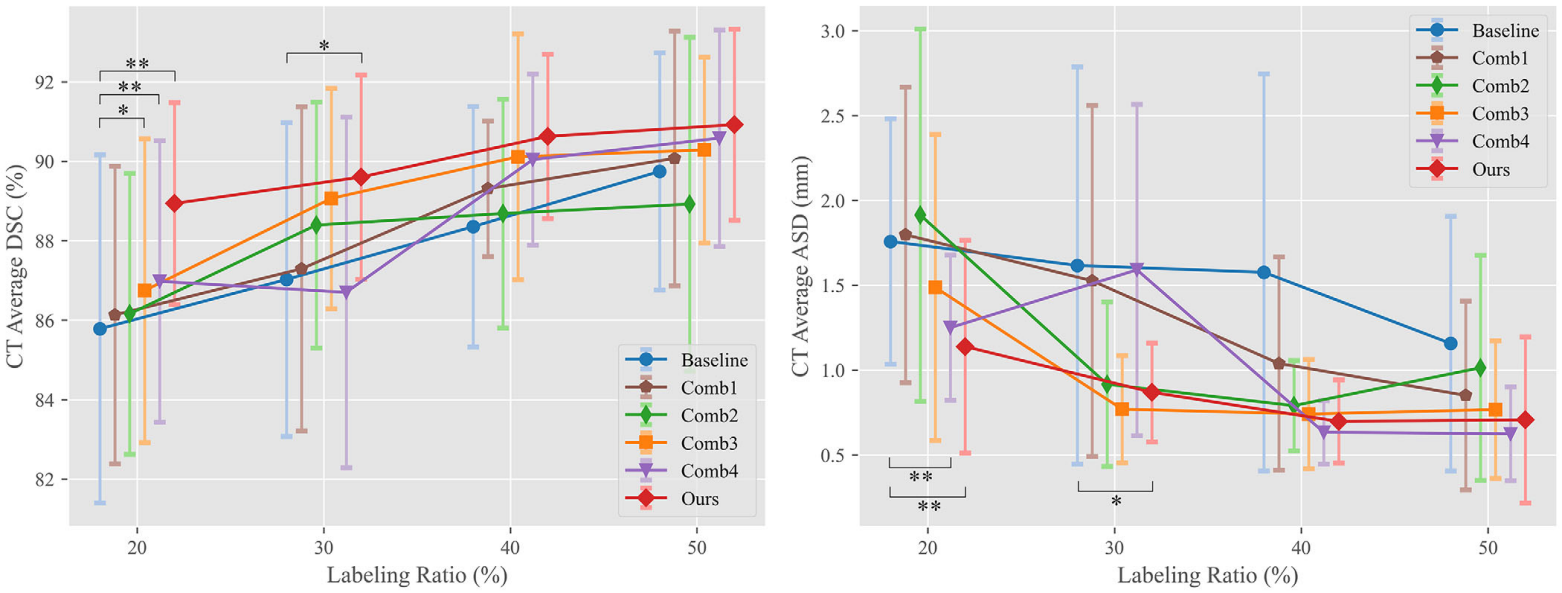
Visualization of the effectiveness of key components in the proposed across variable target domain labeled ratios with 95% confidence interval. **Indicate *p*‐value ≤ 0.05 from two‐sided paired *t*‐test when comparing baseline with other methods. *Indicate *p*‐value ≤ 0.10 from two‐sided paired *t*‐test when comparing baseline with other methods.

To further demonstrate the efficacy of individual components within our model for abdominal multi‐organ segmentation, exemplified by MR‐CT domain adaptation, we present a comparative analysis of the proposed approach (which denoted Ours) and four types of combinations (which denoted Comb1‐Comb4) against the supervised Baseline (which is the same as the DSBN method in Table 1) across varying labeled data ratios within the target domain, as delineated in Table 3. Two methods, excluding the SE‐MT (Baseline and Comb4), employ a joint‐training strategy, which is the SDA approach without utilizing $T_{U}$. Two‐sided paired *t*‐tests were conducted on the average results across four organs to compare the baseline with other methods at different labeled ratios (20%, 30%, 40%, and 50%). Overall, the proposed method achieves statistically significant improvements compared to the baseline with a 3.17% increase in DSC (*p* ≤ 0.05) and a 0.63 mm decrease in ASD (*p* ≤ 0.05) when only 20% of the target domain data was labeled. As the proportion of labeled data increases to 30%, 40%, and 50%, the statistical significance diminishes, but the mean improvements continue to decrease. This indicates that the proposed method is particularly effective in scenarios where labeled data is scarce. In contrast, Comb1, which is the SE‐MT method in Tables 1 and 2, leverages $T_{U}$ and obtains improvements over the baseline in DSC by 0.35%, 0.27%, 0.96%, and 0.33% with target domain labeled ratios of 20%, 30%, 40%, and 50%, respectively. This underscores the SE‐MT structure's capacity to enhance the network's segmentation performance. A small improvement is obtained by adding the GLF module, and this is called Comb2. Based on the SE‐MT structure, the addition of SAD modules in the encoder of the semantic segmentation network, which is named as Comb3, significantly improves performance compared to the baseline when only 20% of the target domain data was labeled. Integrating the SAD module into the standard UNet‐based SE‐MT structure results in a 0.61% improvement in the DSC and a 0.31 mm decrease in the ASD compared to the SE‐MT structure alone on the 20% target domain labeled ratio. Furthermore, we respectively show the DSC and ASD scores of our results in Figure 7 (left) and Figure 7 (right) to clearly demonstrate the performance differences. These findings demonstrate a significant improvement in performance using our method, especially when dealing with a scarcity of labeled data. This confirms the effectiveness of our model in the SSDA framework for abdominal multi‐organ semantic segmentation.

**TABLE 3:** Ablation study of key components.

		Key components						
Labeled ratio	Settings	GLF	SAD	SE‐MT	DSC (%)	*p*‐values	ASD (mm)	*p*‐values
20%	Baseline	x	x	x	85.78 ± 5.48	–	1.76 ± 0.81	–
	Comb1	x	x	√	86.13 ± 4.68 (↑0.35)	7.47E‐01	1.80 ± 1.09 (↑0.04)	8.00E‐01
	Comb2	√	x	√	86.16 ± 4.42 (↑0.38)	6.05E‐01	1.91 ± 1.37 (↑0.15)	6.81E‐01
	Comb3	x	√	√	86.74 ± 4.78 (↑0.96)	9.90E‐02	1.49 ± 1.13 (↓0.27)	3.92E‐01
	Comb4	√	√	×	86.98 ± 4.43 (↑1.20)	3.00E‐02	1.25 ± 0.80 (↓0.51)	3.70E‐02
	Ours	√	√	√	88.95 ± 3.17 (↑3.17)	1.70E‐02	1.13 ± 0.89 (↓0.63)	3.80E‐02
30%	Baseline	x	x	x	87.02 ± 4.94	–	1.62 ± 1.46	–
	Comb1	x	x	√	87.29 ± 5.09 (↑0.27)	8.20E‐01	1.53 ± 1.29 (↓0.09)	7.57E‐01
	Comb2	√	x	√	88.39 ± 3.87 (↑1.37)	2.29E‐01	0.92 ± 0.61 (↓0.70)	1.14E‐01
	Comb3	x	√	√	89.06 ± 3.46 (↑2.04)	2.49E‐01	0.77 ± 0.39 (↓0.85)	1.70E‐01
	Comb4	√	√	x	86.70 ± 5.51 (↓0.32)	8.31E‐01	1.59 ± 1.22 (↓0.03)	9.69E‐01
	Ours	√	√	√	89.60 ± 3.22 (↑2.58)	9.30E‐02	0.87 ± 0.54 (↓0.75)	8.30E‐02
40%	Baseline	x	x	x	88.35 ± 3.78	–	1.58 ± 1.46	–
	Comb1	x	x	√	89.31 ± 2.13 (↑0.96)	3.58E‐01	1.04 ± 0.79 (↓0.54)	4.78E‐01
	Comb2	√	x	√	88.69 ± 3.59 (↑0.34)	7.30E‐01	0.79 ± 0.33 (↓0.79)	1.96E‐01
	Comb3	x	√	√	90.11 ± 3.87 (↑1.76)	1.51E‐01	0.74 ± 0.40 (↓0.84)	2.39E‐01
	Comb4	√	√	×	90.04 ± 2.69 (↑1.69)	4.75E‐01	0.63 ± 0.23 (↓0.95)	1.46E‐01
	Ours	√	√	√	90.63 ± 2.59 (↑2.28)	3.20E‐01	0.70 ± 0.31 (↓0.88)	1.59E‐01
50%	Baseline	x	x	x	89.74 ± 3.73	–	1.16 ± 0.94	–
	Comb1	x	x	√	90.07 ± 4.01 (↑0.33)	7.88E‐01	0.85 ± 0.70 (↓0.31)	3.87E‐01
	Comb2	√	×	√	88.92 ± 5.25 (↓0.82)	3.97E‐01	1.01 ± 0.83 (↓0.15)	1.55E‐01
	Comb3	x	√	√	90.28 ± 2.92 (↑0.54)	4.72E‐01	0.77 ± 0.51 (↓0.39)	1.08E‐01
	Comb4	√	√	x	90.58 ± 3.40 (↑0.84)	1.68E‐01	0.62 ± 0.35 (↓0.54)	1.02E‐01
	Ours	√	√	√	90.92 ± 3.00 (↑1.18)	2.67E‐01	0.71 ± 0.61 (↓0.45)	2.44E‐01

Abbreviations: ASD, average symmetric surface distance; DSC, dice similarity coefficient; SAD, domain‐specific batch normalization; GLF, global‐local fusion; SE‐MT, self‐ensembling mean‐teacher.

#### 3.2.2 Hyperparameter selection

Additionally, we evaluated the performance of our model using various pairs of dilation rates, specifically 1&3, 1&5, 1&7, 3&5, 3&7, and 5&7. As shown in Table 4, the combination of dilation rates 1 and 3 outperformed the other pairs in both DSC and ASD score. Two‐sided paired *t*‐tests indicate that the combination for dilation rates of 1&3 achieves significantly better results compared to other combinations in most cases at the significant level of 0.1.

**TABLE 4:** Comparison with different combination of dilation rates with 20% labeled target domain images.

	BTCV→CHAOS				CHAOS→BTCV			
	DSC (%)	*p*‐values	ASD (mm)	*p*‐values	DSC (%)	*p*‐values	ASD (mm)	*p*‐values
**1&3**	88.97 ± 1.33	–	1.12 ± 0.49	–	88.95 ± 3.17	–	1.13 ± 0.89	–
1&5	87.37 ± 2.05	5.50E‐02	2.70 ± 2.44	2.00E‐03	85.79 ± 5.48	3.70E‐02	1.63 ± 1.37	7.00E‐03
1&7	87.15 ± 2.51	5.60E‐02	2.93 ± 3.52	6.40E‐02	86.45 ± 4.99	2.40E‐02	1.15 ± 0.68	1.29E‐01
3&5	87.91 ± 1.89	7.50E‐02	2.68 ± 2.77	3.30E‐02	86.19 ± 4.74	4.60E‐02	1.47 ± 1.24	1.30E‐02
3&7	88.43 ± 1.30	5.36E‐01	2.93 ± 3.46	6.10E‐02	86.24 ± 4.78	4.80E‐02	1.30 ± 0.83	8.70E‐02
5&7	88.35 ± 1.05	5.12E‐01	2.90 ± 1.97	1.00E‐03	86.50 ± 5.16	5.00E‐02	1.31 ± 1.09	9.20E‐02

Abbreviations: ASD, average symmetric surface distance; DSC, dice similarity coefficient.

In Equation (12), the parameter λ plays a crucial role in balancing the impact of consistency loss and supervision loss on the network's parameters. A too‐high value of λ can introduce excessive erroneous information, potentially causing the training process to fail. Conversely, a too low value limits the model's ability to effectively leverage semantic information from unlabeled data during training. To optimize the selection of this critical trade‐off weight λ, we conducted comprehensive experiments, keeping the weights for dice loss and cross‐entropy loss fixed at 0.7 and 0.3, respectively. We evaluated the DSC and ASD performance across different λ values set at 0.1, 0.5, 1.0, 2.0, and 3.0. According to the results presented in Table 5, setting λ to 2.0 provides the best performance on the abdominal multi‐organ CT dataset. Two‐sided paired *t*‐tests were performed on the average results across four organs to compare λ = 2.0 with other parameter settings. The analysis shows that λ = 2.0 achieves significantly higher DSC than most other parameter settings, with *p*‐values ≤ 0.1. This setting enables our proposed model to maximally benefit from the unlabeled datasets while preventing training failures. The value of unlabeled data in the target domain is underscored, emphasizing the need for careful consideration in our analysis.

**TABLE 5:** Comparison with different trade‐off weights *λ* with 20% labeled target domain (CT) images.

λ	DSC (%)	*p*‐values	ASD (mm)	*p*‐values
0.1	88.66 ± 2.56	9.60E‐02	0.90 ± 0.32	4.47E‐01
0.5	88.51 ± 3.31	4.70E‐02	0.96 ± 0.49	6.38E‐01
1.0	88.34 ± 3.59	4.20E‐02	1.14 ± 0.78	9.81E‐01
**2.0**	88.95 ± 3.17	–	1.13 ± 0.89	–
3.0	88.51 ± 2.36	5.00E‐02	1.06 ± 0.79	8.65E‐01

Abbreviations: ASD, average symmetric surface distance; DSC, dice similarity coefficient.

To further examine the impact of the numerical values of $\lambda_{dice}$ and $\lambda_{ce}$ within the supervised segmentation loss function on the outcomes, we conduct additional experiments. The results of these experiments are presented in Table 6. In our experimental setup, the sum of $\lambda_{dice}$ and $\lambda_{ce}$ equals 1, with the best performance observed when $\lambda_{dice}$ is adjusted to 0.7. Two‐sided paired *t*‐tests were performed to compare the combination of $\lambda_{dice}$ = 0.7 & $\lambda_{ce}$ = 0.3 with other parameter combinations based on the average results across four organs. As shown in Table 6, this setting achieves significantly better DSC compared to other parameter settings with *p*‐values ≤ 0.1. Additionally, for ASD, the model performs better with these parameters compared to $\lambda_{dice}$= 0.3, $\lambda_{ce}$= 0.7, and $\lambda_{dice}$= 0.8, $\lambda_{ce}$= 0.2 with *p*‐values ≤ 0.1.

**TABLE 6:** Comparison with different trade‐off weights $\lambda_{\mathrm{dice}}$ and $\lambda_{\mathrm{ce}}$ with 20% labeled target domain (CT) images.

	$\lambda_{ce}$	DSC (%)	*p*‐values	ASD (mm)	*p*‐values
0.3	0.7	86.85 ± 5.29	4.90E‐02	1.29 ± 0.99	6.60E‐02
0.4	0.6	87.05 ± 5.28	4.00E‐02	1.12 ± 0.70	9.51E‐01
0.5	0.5	88.86 ± 3.56	7.90E‐02	0.77 ± 0.37	1.72E‐01
0.6	0.4	88.61 ± 3.30	8.30E‐02	0.84 ± 0.36	4.38E‐01
**0.7**	**0.3**	88.95 ± 3.17	–	1.13 ± 0.89	–
0.8	0.2	87.48 ± 4.46	8.50E‐02	1.25 ± 0.93	7.30E‐02

Abbreviations: ASD, average symmetric surface distance; DSC, dice similarity coefficient.

## 4 CONCLUSION

In this work, we proposed a novel SSDA framework for abdominal multi‐organ segmentation. This method enhances segmentation by modifying the UNet architecture. In the encoder part, we integrated the scale‐aware with domain‐specific batch normalization module into the convolutional blocks for adapting the variations between different imaging domains. In the bottleneck part of the UNet, we developed a GLF module to extract local information and long‐distance dependencies. Furthermore, we seamlessly integrated these components into SE‐MT training framework. These innovations reduce the need for extensive manual annotations and improve segmentation accuracy. The experiments on two public datasets have shown that our proposed model outperformed the state‐of‐the‐art methods. Future work will focus on enhancing segmentation precision through the integration of boundary confidence measures and extending our methodology to various medical imaging segmentation tasks to assess the effectiveness of different confidence estimation strategies.

## AUTHOR CONTRIBUTIONS

Kexin Han, the principal investigator, managed data, developed methodologies, conducted analysis, validated methods, and wrote the first draft. Qiong Lou, provided resources, verified findings, and ensured project oversight. Fang Lu led and coordinated this study. She also supplied resources, performed statistical analysis, and refined the manuscript's final draft. Both Qiong Lou and Fang Lu provided funding for this research. All authors reviewed and approved the final manuscript.

## CONFLICT OF INTEREST STATEMENT

The authors declare no conflicts of interest.

## Data Availability

The dataset utilized in this study is publicly accessible. The data can be obtained from the following source: (1) BTCV dataset (Multi‐Atlas Labeling Beyond the Cranial Vault): https://huggingface.co/datasets/jiayuanz3/btcv/resolve/main/btcv.zip. (2) CHAOS dataset (CHAOS—Combined (CT‐MR) Healthy Abdominal Organ Segmentation Challenge Data): https://zenodo.org/records/3431873.
